# Vegetarian diet, change in dietary patterns, and diabetes risk: a prospective study

**DOI:** 10.1038/s41387-018-0022-4

**Published:** 2018-03-09

**Authors:** Tina H. T. Chiu, Wen-Harn Pan, Ming-Nan Lin, Chin-Lon Lin

**Affiliations:** 1 Department of Nutrition Therapy, Dalin Tzu Chi Hospital, Buddhist Tzu Chi Medical Foundation. No. 2, Min-Sheng Road, Dalin Town, Chiayi County, 622 Taiwan; 2 Graduate Institute of Epidemiology and Preventive Medicine, National Taiwan University, No. 17, Xu-Zhou Road, Taipei, 100 Taiwan; 3 Department of Medicine, College of Medicine, Tzu Chi University, Hualien, Taiwan. No.701, Sec. 3, Chung Yang Road, Hualien, 970 Taiwan; 4 Institute of Biomedical Sciences, Academia Sinica, Address: 128 Sec. 2, Academia Road, Nankang, Taipei, 115 Taiwan; 5 Department of Family Medicine, Dalin Tzu Chi Hospital, Buddhist Tzu Chi Medical Foundation. No. 2, Min-Sheng Road, Dalin Town, Chiayi County, 622 Taiwan; 6 Department of Family Medicine, College of Medicine, Tzu Chi University, Hualien, Taiwan. No.701, Sec. 3, Chung Yang Road, Hualien, 970 Taiwan; 7 Department of Internal Medicine, Hualien Tzu Chi Medical Center, Hualien, Taiwan. No. 707, Sec. 3, Chung Yang Road, Hualien, 970 Taiwan; 8 Department of Internal Medicine, College of Medicine, Tzu Chi University, Hualien, Taiwan. No.701, Sec. 3, Chung Yang Road, Hualien, 970 Taiwan

## Abstract

**Background/objectives:**

Vegetarian diets are inversely associated with diabetes in Westerners but their impact on Asians—whose pathophysiology differ from Westerners—is unknown. We aim to investigate the association between a vegetarian diet, change in dietary patterns and diabetes risk in a Taiwanese Buddhist population.

**Methods:**

We prospectively followed 2918 non-smoking, non-alcohol drinking Buddhists free of diabetes, cancer, and cardiovascular diseases at baseline, for a median of 5 years, with 183 incident diabetes cases confirmed. Diet was assessed through a validated food frequency questionnaire at baseline and a simple questionnaire during follow-ups. Incident cases of diabetes were ascertained through follow-up questionnaires, fasting glucose and HbA1C. Stratified Cox Proportional Hazards Regression was used to assess the effect of diets on risk of diabetes.

**Results:**

Consistent vegetarian diet was associated with 35% lower hazards (HR: 0.65, 95% CI: 0.46, 0.92), while converting from a nonvegetarian to a vegetarian pattern was associated with 53% lower hazards (HR: 0.47, 95% CI: 0.30, 0.71) for diabetes, comparing with nonvegetarians while adjusting for age, gender, education, physical activity, family history of diabetes, follow-up methods, use of lipid-lowering medications, and baseline BMI.

**Conclusion:**

Vegetarian diet and converting to vegetarian diet may protect against diabetes independent of BMI among Taiwanese.

## Introduction

The rapid growth of diabetes creates tremendous health and economic burdens worldwide^1^. In Taiwan, diabetes patients incur 2.8 times more medical expense than matched non-diabetes individuals, and used up 29% of total healthcare expenditure^2^, while high blood glucose accounts for the highest adult mortality among major metabolic, lifestyle, and environmental factors^3^. Preventive strategies are desperately needed, particularly in Asia, where the largest number of diabetes cases is expected^1^, and diabetes tend to occur despite lower BMI^4^.

Diabetes is driven by impaired insulin action (insulin resistance) and insulin secretion (islet dysfunction and incretin failure), co-influenced by genetics and environment^5^. While diabetes in Caucasian is highly attributed to obesity and insulin resistance, emerging evidence suggests that β-cell dysfunction may be more predictive of diabetes in East Asians^6^. In fact, Japanese with normal glucose tolerance already showed an insulin secretion ability similar to Caucasian diabetes patients^7^. The potential pathophysiological difference warrants research on population-specific preventive strategies.

Vegetarian diets^8^ and plant-based dietary patterns^9^ have been shown to dramatically reduce diabetes risks in American populations, but their effect in Asians is less clear. Unlike in Westerns, the effect of meat and fish on diabetes is inconsistent in Asians^10–13^, and may be modified by BMI^12, 14^. Whether complete avoidance of meat and fish is necessary to boost protection for diabetes remains puzzling, as Asians tend to be leaner and consume less meat than Westerners. Our primary aim is to examine the association between a vegetarian diet, converting from a nonvegetarian to a vegetarian dietary pattern, and diabetes risk in a prospective cohort study. Our secondary aim is to explore potential interaction between dietary patterns and baseline metabolic status (BMI, metabolic syndrome, and other metabolic characteristics).

## Methods

### Study design and population

The Tzu Chi Health Study (TCHS) is a prospective cohort study that recruited 4625 Tzu Chi volunteers (age 18 to 87 years) from 2007 to 2009 at the Buddhist Dalin Tzu Chi Hospital. Tzu Chi volunteers are devoted Buddhists who volunteer regularly for the Buddhist Tzu Chi Foundation for a variety of charity works and disaster relief. Many nonvegetarian volunteers converted to vegetarian in the year 2011 due to a religious event. Because the nonvegetarians consumed only small amount of meat (median intake per day, women: 10 g, men: 22 g) and fish (median intake per day, women: 6 g, men: 15 g)^15^, this cohort is equipped with a unique opportunity to distinguish disease risk between true vegetarians and those with little to modest meat consumption.

Participants were followed from 2010 to 2012 (first follow-up), and from 2013 to mid-2016 (second follow-up). Every three years, a post-card was sent to invite each participant for a follow-up health examination during which participants also fill out a questionnaire on disease diagnosis. Participants who did not return for health examination by the end of 2015 were sent a follow-up questionnaire in May 2016 to assess their dietary practices and disease conditions (for each disease, choices includes: ‘no’, ‘yes’, ‘not sure’, and the time of diagnosis). If the questionnaire was not returned within a month, a research assistant called the participants to interview this questionnaire. The study was approved by the Institutional Review Board for ethics in the Buddhist Dalin Tzu Chi Hospital. All participants gave written informed consents. The study has been registered at ClinicalTrials.gov (ID: NCT: 03204552).

### Assessment of metabolic characteristics and covariates

At baseline, trained research assistants interviewed all participants on basic demographics, medical history and relevant medications, family history of diseases, diet, use of cigarette and alcohol, and leisure time physical activities (LTPA). Fasting glucose and blood lipids, including triglyceride (TG), high density lipoprotein cholesterol (HDL-C) and other cholesterol were assessed by the Integra 800 system (Roche Diagnostics, Indianapolis, IN) at baseline and Dimension RxL Max (Siemens Healthcare Diagnostics Inc, Newark, NJ) at follow-ups. HbA1C was assessed by Variant Turbo (BIO-RAD, Hercules, CA). Heights and weights were measured using an electronic scale while participants stood in an upright position without shoes. Waist circumference was measured at navel. Blood pressures were assessed using VP1000 system (Colin, Komaki, Japan). Metabolic syndrome was defined by the National Cholesterol Education Program, Adult Treatment Panel (ATP III) but using the Taiwanese cut points for waist circumference (≥90 cm for men, ≥80 cm for women). Fatty liver was assessed using ultrasonography by hospital gastroenterologists.

### Assessment of diet

At baseline, participants were interviewed on a 64-item quantitative food frequency questionnaire (FFQ) that had been validated within our cohort participants, and showed good validity and reliability when compared with repeated dietary records and biomarkers^16^. At follow-ups, all participants answered a simple questionnaire that asked whether they are vegetarians (choices including: not vegetarian, breakfast vegetarian, vegetarian on 1st and 15th day of each lunar month (a practice for many Buddhists), irregular dates of vegetarian diets, full time vegetarian), and the type of vegetarian diet (vegan, lacto-ovo vegetarian, lacto-vegetarian, ovo-vegetarian). Only full-time vegetarians were considered vegetarians in our analysis.

Dietary patterns are divided into 4 types: (1) vegetarians are defined as those who have been following vegetarian diets at baseline and all the follow-ups; (2) the reverted are those who were initially vegetarians but became nonvegetarians at one of the follow-ups; (3) the converted are those who were initially nonvegetarians but became vegetarians later; and (4) the nonvegetarians are those who had consistently reported eating nonvegetarian diet in baseline and follow-ups. Diets assessed after diabetes diagnosis were discarded from the analysis.

### Case ascertainment

Diabetes cases were identified if participants reported diabetes diagnosis at follow-up questionnaires, or if their HbA1C ≥6.5%. Participants with only one fasting blood glucose ≥7.0 mmol/L but otherwise normal HbA1C were identified as possible diabetes cases. For these individuals, a physician coauthor (MNL) further reviewed their medical records to check if they have additional blood tests or prescription of diabetes medication to determine their diabetes status. Participants without further tests or available medical records were considered unconfirmed diabetes events (*n* = 25) and were excluded, but included as cases in a sensitivity analysis.

### Statistical analysis

Baseline characteristics between different dietary patterns were compared using ANOVA test (for continuous variables) and Chi-square test (for categorical variables).

Cox regression was used to analyze the association between dietary patterns and risk of diabetes, with stratification for LTPA and follow-up methods (questionnaire only vs health examination) as the interaction term of time with these variables violated proportional hazard assumption. Model 1 adjusted for age, sex, education, family history of diabetes, LTPA, lipid-lowering medication, methods of follow-up. Model 2 additionally adjusted for BMI to estimate the protective association independent of BMI (a mediator). Time of disease occurrence was set to be the time that the first abnormal glucose was identified (HbA1c ≥6.5% or fasting blood glucose ≥7.0 mmol/L). For participants who reported diagnosis of diabetes at questionnaire but could not remember the time of diabetes diagnosis, censor time was set to be half-way between the previous known disease-free time point and the follow-up time in which diabetes was reported. For those who did not report having diabetes in the questionnaire, but were found to have diabetes during health examination, the date of health examination was used as the date of disease onset. Subgroup analyses by gender, baseline metabolic syndrome, fatty liver, BMI (<24 or ≥24 kg/m^2^), fasting glucose (<5.56 or ≥5.56 mmol/L), TG (<1.69 or ≥1.69 mmol/L), and HDL-C (men: <1.03 or ≥1.03 mmol/L, women: <1.29 or ≥1.29 mmol/L) levels were performed. Test of interaction was performed by including a cross-product term in the regression model.

Several sensitivity analyses were performed: (1) 25 unconfirmed diabetes event were treated as diabetes cases. (2) To ensure our result was not affected by detection bias from different follow-up methods (health examination vs questionnaire-only), we performed another sensitivity analysis in which only self-reported diabetes were counted as cases. (3) We adjusted for metabolic syndrome in addition to Model 2. (4) Age was used as the underlying time scale for Cox regression, with left truncation at study entry. All analysis were conducted using SAS Statistical Software (version 9.4, SAS Institute, Cary, NC).

## Results

### Exclusion and loss to follow-up

To calculate the incidence of diabetes and avoid bias related to comorbidities, we excluded participants with baseline diabetes or fasting blood glucose ≥7.0 mmol/L (*n* = 322), reported history of cancer (*n* = 172), coronary heart disease (*n* = 194), stroke (*n* = 26). We also excluded those who have ever used cigarette (*n* = 691) or alcohol (*n* = 606), as cigarette smoking may modify the effect of meat on diabetes^17^ and alcohol is frequently associated with smoking. This left a total of 3185 participants. We further excluded those lost to follow-up (*n* = 210), those who did not know about their diabetes status in follow-up questionnaire (*n* = 8), those missed the item on diabetes diagnosis in the questionnaire (*n* = 42, likely without diabetes as they responded to a few diseases they had a diagnosis and left all other diseases blank), and unconfirmed diabetes events (*n* = 25, some may be included as non-cases up until the time that normal blood glucose and HbA1C were observed), leaving a total of 2918 participants in the final analysis. A flow chart summarizing number of participants at each stage is available at Supplemental Figure.

Of those who were followed through health examination, 1902 returned for first follow-up (2010 to 2012), 1739 returned for second follow-up (2013 to mid-2016), and 1247 returned for both. The baseline characteristics by follow-up status and methods were compared at Supplemental Table 1. Men were less likely to be followed (either through health examination or mailed questionnaire), while women with lower education were more likely to return for health examination. No significant difference in diet, lifestyle and metabolic risk factors were observed among those returned for health examination, those responded to the follow-up questionnaire, and those lost to follow-up.

### Baseline characteristics

Table 1 shows the baseline characteristics of different dietary patterns. Nonvegetarians tend to have higher BMI, waist circumference (among female), and fasting blood glucose. Female were more likely to consume vegetarian diets at baseline or switch to a vegetarian diet later. The converted had the lowest proportion with metabolic syndrome and elevated TG.

**Table 1:** Baseline characteristics of participants with different dietary patterns

	Vegetarian	Reverted	Converted	Nonvegetarian	P
*n*	1053	124	697	1044	
Age, years	54.1 (9)	53.6 (8.5)	52.6 (8.7)	52.7 (9.8)	0.001
BMI, kg/m^2^	22.8 (2.8)	23.2 (3.4)	23.3 (3.1)	23.8 (3.3)	<.001
Waist (all), cm	74.6 (7.8)	75.7 (9.2)	75.5 (8)	77.4 (8.8)	<.001
Female*	73.5 (7.3)	74.6 (9.4)	74.1 (7.6)	74.8 (7.8)	0.011
Male**	80.7 (7.3)	81.3 (6.2)	80.6 (7.2)	82 (8.4)	0.16
Weight (all), kg	56.6 (8.3)	57.6 (9.2)	58.7 (9.1)	61.2 (10.4)	<.001
Female*	55.1 (7.4)	55.9 (8.8)	56.9 (8.3)	57.6 (8.4)	<.001
Male**	64.1 (8.6)	65.9 (6)	65.6 (8.4)	67.5 (10.5)	0.002
Height (all), cm	158 (6)	157 (7)	159 (7)	160 (8)	<.001
Female*	156 (5)	156 (5)	157 (6)	156 (6)	0.09
Male**	166 (5)	167 (6)	167 (6)	167 (6)	0.26
Fasting glucose, mg/dL	90 (8)	92 (8)	91 (9)	92 (9)	<.001
Female, %	84	83	79	63	<.001
Education, %					
Elementary	28	27	22	23	0.003
Secondary	52	56	55	51	
College	20	17	24	26	
Education (female*), %					
Elementary	30	29	24	26	0.22
Secondary	53	55	56	55	
College	17	16	20	19	
Education (male**), %					
Elementary	19	19	14	17	0.79
Secondary	46	57	50	45	
College	36	24	35	38	
LTPA, min/week					
<30	38	45	36	35	0.09
30–180	33	30	35	33	
>180	29	25	28	33	
LTPA (female*), min/week					
<30	39	48	38	38	0.62
30–180	33	28	35	35	
>180	28	24	27	28	
LTPA (male**), min/week					
<30	35	33	30	29	0.25
30–180	31	38	37	29	
>180	34	29	33	42	
Family history of diabetes, %	27	26	29	31	0.18
Follow-up methods					
Health examination	84	79	89	75	<.001
Questionnaire only	16	21	11	25	
Metabolic syndrome, %	14	17	10	15	0.035
Fatty liver, %	49	53	50	56	0.008
Impaired fasting glucose, %	11	15	14	17	0.001
BMI categories, %					
<18.5	5	4	3	2	<.001
18.5–23.9	65	58	60	55	
24.0–26.9	23	27	28	27	
≥27.0	7	10	9	15	
Elevated TG, %	17	20	13	17	0.026
Low HDL-C, %	38	29	26	24	<.001
Use of lipid medication, %	1.6	2.4	2.0	3.1	0.15

*P*-values are from ANOVA and *X*^2^ test. Reverted, diet changed from vegetarian to nonvegetarian; converted, diet changed from nonvegetarian to vegetarian

*BMI* body mass index, *LTPA* leisure time physical activities, *HDL-C* high-density lipoprotein cholesterol

*Women: 886 vegetarians, 103 reverted, 550 converted, 660 nonvegetarians

**Men: 167 vegetarians, 21 reverted, 147 converted, 384 nonvegetarians

### Dietary patterns and diabetes risks

In the median 5.2 years of follow-up, 183 individuals developed incident diabetes. The association between dietary patterns and diabetes in all participants are shown in Table 2. Consistent vegetarians and the converted showed about 40–60% reduction in risk of diabetes, compared with nonvegetarians. Those reverting from vegetarian diet to nonvegetarian diet (Reverted) also experienced a lower risk of diabetes compared with nonvegetarians (as indicated by the magnitude of hazard ratio) but the association did not reach statistical significance due to small sample size (only 6 cases in 583 person-years of follow-up).

**Table 2:** Hazard ratios (95% confidence intervals) of diabetes by different dietary patterns

	Vegetarian	Reverted	Converted	Nonvegetarian
No/person-year	55/5431	6/583	29/3496	93/5456
Crude	0.59 (0.42, 0.82)	0.58 (0.25, 1.32)	0.48 (0.32, 0.73)	1 (Ref)
Model 1	0.54 (0.38, 0.76)	0.60 (0.26, 1.37)	0.45 (0.29, 0.68)	1 (Ref)
Model 2	0.65 (0.46, 0.92)	0.63 (0.27, 1.45)	0.47 (0.30, 0.71)	1 (Ref)

Reverted, diet changed from vegetarian to nonvegetarian. Converted, diet changed from nonvegetarian to vegetarian. Data are hazard ratios (and 95% CI) estimated using Cox regression stratified by follow-up methods and leisure time physical activities. Model 1 adjusted for age, gender, education, leisure time physical activities, family history of diabetes, follow-up methods (health examination or questionnaire only), and lipid medication. Model 2 additionally adjusted for BMI

Additional subgroup analyses and the test for interactions are presented in Table 3. All the analysis in this table were adjusted for BMI (same as Model 2 in Table 2). The association between dietary patterns and diabetes did not differ significantly between subgroups by gender, metabolic syndrome, fasting glucose, and HDL at baseline, as indicated by the non-significant test of interactions and similar trends and magnitudes in hazard ratios. The test of interaction between dietary patterns and TG was significant (*P*-interaction = 0.039) and that the hazard ratio of diabetes in vegetarians and the converted (vs nonvegetarians) are significant mainly in those with normal TG but not those with elevated TG. Overall, converting to vegetarian is strongly associated with protection among those with healthier baseline metabolic characteristics (normal TG: HR = 0.32, without metabolic syndrome: HR = 0.40, and BMI < 24: HR = 0.24). Due to limited case numbers in the reverted, all the results in the subgroup analysis for the reverted (versus nonvegetarians) were statistically insignificant and some subgroup analyses were not possible (data not shown).

**Table 3:** Subgroup analysis of dietary patterns and diabetes risk by gender and metabolic characteristics

Subgroups	Vegetarian	Converted	Nonvegetarian	P-interaction
*Gender*				0.35
Female	0.67 (0.45, 0.99)	0.41 (0.25, 0.68)	1 (Ref)	
Male	0.56 (0.24, 1.31)	0.73 (0.32, 1.71)	1 (Ref)	
*Metabolic syndrome*				0.67
No	0.66 (0.42, 1.04)	0.40 (0.22, 0.72)	1 (Ref)	
Yes	0.58 (0.33, 1.02)	0.62 (0.33, 1.18)	1 (Ref)	
*Fatty liver*				0.63
No	1.10 (0.50, 2.42)	0.62 (0.22, 1.79)	1 (Ref)	
Yes	0.54 (0.36, 0.81)	0.48 (0.30, 0.76)	1 (Ref)	
*BMI*				0.23
<24	0.61 (0.35, 1.06)	0.24 (0.10, 0.58)	1 (Ref)	
≥24	0.64 (0.41, 1.01)	0.62 (0.38, 1.02)	1 (Ref)	
*Fasting glucose*				0.85
Normal	0.81 (0.50, 1.33)	0.48 (0.26, 0.90)	1 (Ref)	
IFG	0.64 (0.38, 1.09)	0.51 (0.28, 0.92)	1 (Ref)	
*TG*				0.039
Normal	0.58 (0.38, 0.90)	0.32 (0.18, 0.55)	1 (Ref)	
Elevated	0.71 (0.38, 1.32)	0.91 (0.45, 1.85)	1 (Ref)	
*HDL*				0.77
Normal HDL	0.62 (0.37, 1.04)	0.45 (0.25, 0.80)	1 (Ref)	
Low HDL	0.58 (0.35, 0.96)	0.50 (0.26, 0.94)	1 (Ref)	

All Models adjusted for age, gender, education, leisure time physical activities, family history of diabetes, follow-up methods (health examination or questionnaire only), lipid medication, and BMI (same as Model 2 in Table 2). Converted, diet changed from nonvegetarian to vegetarian; *IFG*impaired fasting glucose, *MS*metabolic syndrome, *TG*triglyceride, *HDL-C*high-density lipoprotein cholesterol. Normal glucose: <5.56 mmol/L; IFG: ≥5.56 mmol/L; normal TG: <1.69 mmol/L; elevated TG: ≥1.69 mmol/L; normal HDL-C: ≥1.03 mmol/L for men, ≥1.29 mmol/L for women; low HDL-C: <1.03 mmol/L for men, <1.29 mmol/L for women. Data are hazard ratios (and 95% CI) estimated using Cox regression stratified by follow-up methods and leisure time physical activities. Model 1 adjusted for age, gender, education, leisure time physical activities, family history of diabetes, follow-up methods (health examination or questionnaire only), and lipid medication. Model 2 additionally adjusted for BMI

### Sensitivity analyses

Our sensitivity analyses (Supplemental Table 2) showed similar findings as our main analysis: (1) When unconfirmed diabetes were counted as diabetes cases, similar result was seen in both consistent vegetarians (Model 1: HR = 0.54, 95% CI = 0.38–0.76; Model 2: HR = 0.65, 95% CI = 0.46–0.92) and the converted (Model 1: HR = 0.45, 95% CI = 0.29–0.68; Model 2: HR = 0.47, 95% CI = 0.30–0.71). (2) When counting only questionnaire-assessed self-reported diabetes as cases, similar trends were found for vegetarian (Model 1: HR = 0.56, 95% CI = 0.35–0.89; Model 2: HR = 0.69, HR = 0.43–1.11), and the converted (Model 1: HR = 0.49, 95% CI = 0.28–0.87; Model 2: HR = 0.50, 95% CI = 0.29–0.89), though with wider confidence intervals due to a smaller case numbers. (3) Addition of metabolic syndrome to Model 2 showed no substantial change to the result for vegetarians (HR: 0.62, 95% CI: 0.44–0.88) and the converted (HR: 0.49, 95% CI: 0.32–0.75). (4) When age was used as the underlying time scale for Cox regression, similar results were found for vegetarians (Model 1: HR = 0.49, 95% CI = 0.34–0.67; Model 2: HR = 0.60, 95% CI = 0.42–0.86) and the converted (Model 1: HR = 0.45, 95% CI = 0.29–0.68; Model 2: HR = 0.43, 95% CI = 0.28–0.66).

## Discussion

In this prospective cohort study, both vegetarian diet and nonvegetarians switching to vegetarian diets are associated with ~50% reduction in risk of diabetes. This trend did not differ across gender, metabolic syndrome, HDL-C, and fasting glucose status. The potential protective effect of converting to vegetarian appeared to be particularly pronounced in those with initially healthier metabolic status. To the best of our knowledge, this is the first study that (1) examined the prospective association between a vegetarian diet and diabetes in high risk Asians, and (2) addressed how switching from a nonvegetarian diet to a vegetarian diet may influences diabetes risk.

### Plant-based diets

The magnitude of protective association between a vegetarian diet and diabetes in our study (HR before BMI adjustment = 0.54, HR after BMI adjustment = 0.65) is comparable to the Adventist Health Study – 2 (AHS-2; lacto-ovo vegetarians: OR before BMI adjustment = 0.46, OR after BMI adjustment = 0.62)^8^, and the three Harvard cohorts of US nurses and health professionals (highest vs lowest deciles of healthy plant-based diet index: HR before BMI adjustment = 0.55, HR after BMI adjustment = 0.66)^9^. In all these studies, BMI appears to account for only a small portion of the protective association, as the results in all these studies are only slightly attenuated after BMI adjustment. The BMI-independent protective effect is also confirmed in a trial using the Mediterranean diet^18^—another healthy plant-based diet. These studies, together, suggest that vegetarian or healthy plant-based diets may exert protective effect beyond just BMI.

Vegetarians in our cohort and in AHS-2 consumed more whole grains and vegetables than nonvegetarians^15, 19^, and these may protect against diabetes through higher fiber and magnesium^20^, as deficiency in magnesium may impair insulin signaling^21^. In addition, soy is a major source of protein for Taiwanese vegetarians, and soy has been shown to improve insulin resistance when replacing meat in randomized controlled trials^22, 23^. Soy and legume are inversely associated with risk of diabetes in a Chinese cohort^24^. A vegetables-fruits-soy dietary pattern is inversely associated with diabetes incidence in Singaporean Chinese^17^.

### Meat and fish

Although the protective association is likely caused by various plant components, it may have resulted partly from the simultaneous elimination of meat, as components of meat and fish may affect both insulin secretion and insulin resistance. Meat is high in saturated fats, which have been shown to trigger human β-cell apoptosis^25^. Fatty acids from meat have been adversely associated with insulin secretion and Disposition Index (β-cell function accounting for insulin sensitivity)^26^. A trial showed that while plant polyphenol improves glucose metabolism, fish omega-3 fatty acids decreases insulin secretion and postprandial GLP-1^27^. Besides insulin secretion, high intakes of animal protein may induce insulin resistance through 3-Hydroxyinsbutyrate (a valine metabolite) and fibroblast growth factor 21^28^. The positive association between animal protein and type 2 diabetes has also been confirmed in a recent prospective cohort study and an updated meta-analysis^29^.

However, the association between meat and diabetes is equivocal among Asian women: meat was not associated with diabetes risk in Japanese women^13^ and was associated with protection among normal weight Chinese women in Shanghai^12^. None of these studies actually included a diet range of complete meat avoidance, and it is possible that even the lowest quantile in these cohorts did not consume low enough meat (and high enough healthy plant foods) to attain protection. The inverse association between meat intake and diabetes in the Shanghai Women’s Health Study may potentially be confounded by unmeasured social economic factors, early life food insecurity, and cohort effect, as the lowest meat consuming quantile appeared to be older and from lower socioeconomic classes^12^. Lower socioeconomic sectors may have experienced early life under nutrition, which could potentially trigger epigenetic changes that induce diabetes risk^30^.

### Potential interactions and effect modifications

In our subgroup analysis by gender, the insignificant association between vegetarian diet and diabetes in the male subgroup was likely due to small sample size. The hazard ratio of 0.56 (clearly trends toward protection) and the insignificant test for interaction (*P*-interaction = 0.35) suggest that the protective association between a vegetarian diet and diabetes is likely similar for both genders. This surmise is consistent with the results reported for healthy plant-based diets in the three Harvard cohorts of US nurses and health professionals^9^.

In the Shanghai Women’s Health Study^12^ and in Japanese Americans within the Multiethnic Cohort^13^, meat or a meat-fat dietary pattern increased diabetes risk only among overweight individuals. In our subgroup analysis, however, the association between a vegetarian diet and diabetes did not differ significantly across categories of BMI or metabolic syndrome. It is possible that the effect of a vegetarian dietary pattern is not due solely to either the minimization of animal product or the higher functional plant ingredients, but the additive effect of both. While meat may be harmful among the overweight, plant components may be beneficial even among the normal weight, thus extending its effect across different weight status. This result is also found in the PREDIMED-Reus Trial, in which a Mediterranean diet—also a plant-based dietary pattern—showed similar protective trends for different categories of BMI (HR = 0.54 for BMI ≤ 30, HR = 0.56 for BMI > 30), and fasting glucose (HR = 0.53 for fasting glucose ≤ 6.1 mmol/L, HR = 0.32 for fasting glucose > 6.1 mmol/L)^18^.

The effect of lifestyle interventions tend to be more obvious for individuals at high risk than those at low risk^31^, and it should be no surprise that lifestyle trials aiming to prevent diabetes tend to focus on high risk individuals^18, 32^. Interestingly, we found that the association between converting to vegetarian diet and diabetes risk to be quite strong for those with supposedly healthier metabolic status: normal TG (HR = 0.32), without metabolic syndrome (HR = 0.32), and BMI < 24 (HR = 0.24). However, our sample size in this subgroup is limited and residual confounding may still exists. Randomized controlled trials are needed to confirm our findings.

### Change in diet

The strong inverse association between converting to vegetarian diet and diabetes suggests that diabetes risk or protection may be influenced by recent diets. In US nurses and health professionals, change in red meat consumption over 4 years has been associated with diabetes risk independent of baseline red meat intake^33^. Trials using vegetarian diets improve glycemic control in weeks^34^. Switching to a complete plant-based diet may increase butyrate production through gut microbiota alteration in as short as 1 day^35^. Butyrate may induce incretin secretion and improve glucose metabolism^36^. The magnitude of protective association of converting to vegetarian diet (53% reduction in diabetes risk compared with a persistent nonvegetarian diet) in our population is comparable to the Diabetes Prevention Trial (58% reduction in diabetes risk through lifestyle changes aiming at weight reduction), which is more effective than use of metformin (31% reduction) among high risk individuals^32^. These findings, together, suggest that a powerful diabetes preventive potential may be achieved by either a healthy plant-based diet (calorically non-restrictive) or a BMI-lowering intervention aiming to reduce calories and increase physical activities, offering alternatives strategies for both lean (weight loss may lead to undesirable side effects associated with underweight) and obese individuals.

### Study strength and limitations

The prospective design with high follow-up rate (93%) reduces recall and selection biases. The majority (75%) of participants have their diabetes status confirmed by blood tests, which minimize potential undiagnosed diabetes and reduce outcome misclassification. The homogenous population of non-smokers and non-alcohol drinkers from the same religious community may reduce unmeasured confounding and strengthen internal validity, though the generalizability to other population requires further confirmation. Although we attempted to adjust for potential confounders, some residual confounding effect may still remain: (1) Measurement of physical activities through questionnaire may not be accurate or adequate^37^. (2) Conversion to vegetarian diet may be associated with religious devotion and it is unknown whether religious devotion would impact diabetes risk. In addition, the lack of detail in the follow-up questionnaire prevented us from analyzing detail dietary changes—other than meat and fish intake went from small to zero for the converted. Although imperfect, our verification of dietary pattern at follow-ups allowed us to capture dietary changes pertinent to our study aim (vegetarian vs nonvegetarian dietary patterns), providing more insights than most cohorts that rely solely on one baseline diet assessment. Finally, our sample size may be inadequate in some of the subgroups to draw conclusions on potential effect modification by metabolic status. Nevertheless, our finding shows no harm associated with a vegetarian diet in any subgroup and that vegetarian diets could be safely practiced.

Our study suggests that a vegetarian diet may immensely reduce the risk of diabetes in lean Asian populations. Further researches on how animal and plant components affect β-cell function and insulin resistance will be needed to establish the ideal diet for diabetes prevention. Our consistent finding with Western populations has a far-reaching public health and environmental implication. The large and consistent protective association between plant-based diets and diabetes, and the over-consumption of meat by the majority today, suggest enormous population-attributable protection potential of a vegetarian diet. At the same time, shifting toward a plant-based diet is estimated to reduce food-related greenhouse gas emissions by 29–70%^38^. A vegetarian diet may be a stunning solution to the diet–environment–health trilemma that our globe urgently need to tackle, for the welfare, if not the survival, of many who are deeply threatened by climate change and diabetes.

## Electronic supplementary material

Supplemental material
