# Supplementary material for: Vegetarian diet, change in dietary patterns, and diabetes risk: a prospective study
Source: Nutr Diabetes. 2018 Mar 9;8:12. doi: 10.1038/s41387-018-0022-4 (PMC5856738; doi:10.1038/s41387-018-0022-4)
Supplement: Supplementary file 1 — Supplemental material [file 41387_2018_22_MOESM1_ESM.docx]

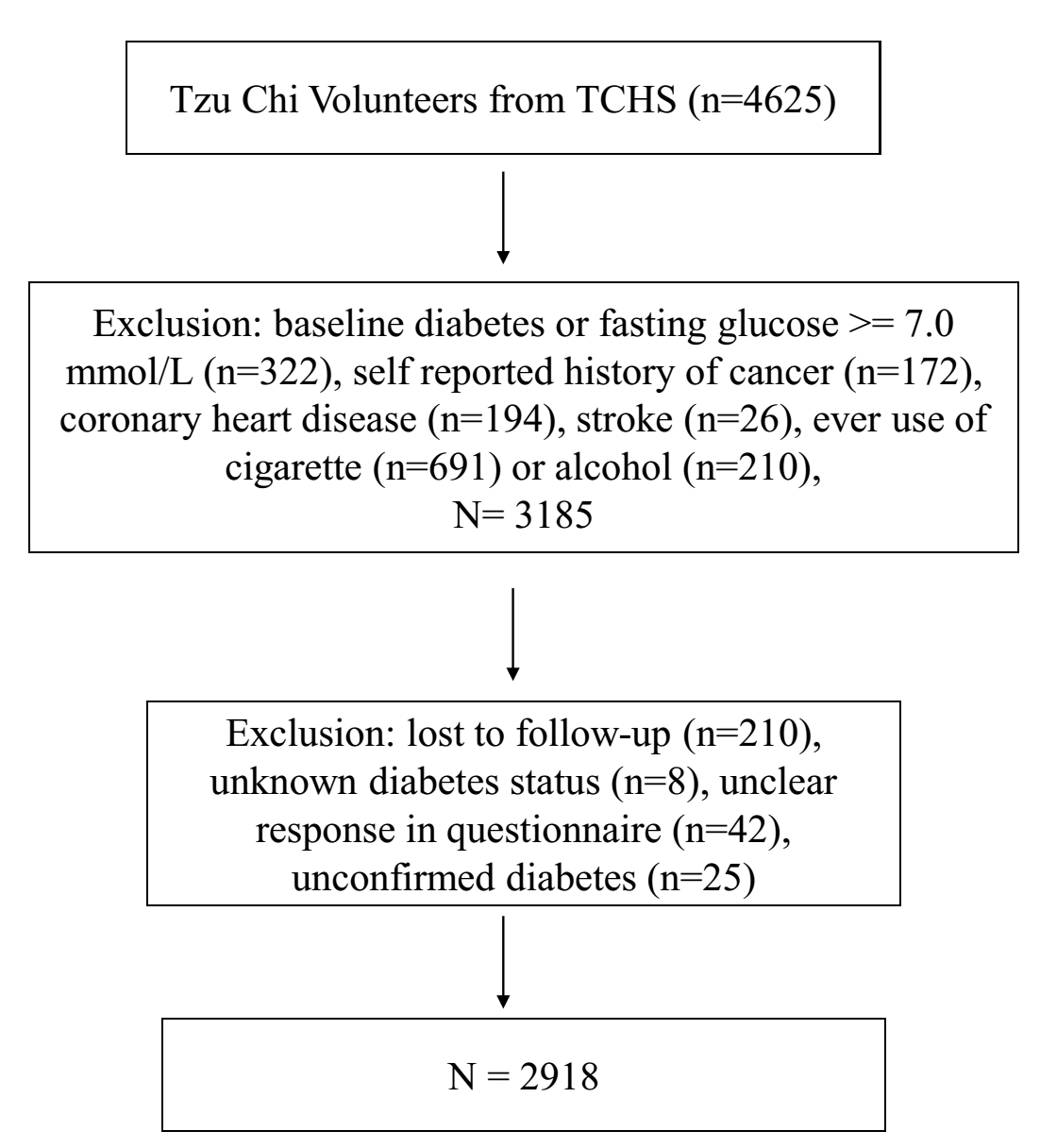


**Supplemental Figure. Number of participants at each exclusion stages.**

**Supplemental Table 1. Baseline characteristics of participants by follow-up status**

|  | Lost to follow-up | Health examination | Questionnaire only | P-value |
| --- | --- | --- | --- | --- |
|  | 210 | 2394 | 581 |  |
| Age | 52.4 (13.2) | 53.7 (8.8) | 51.6 (11) | <.001 |
| BMI | 23 (3.2) | 23.2 (3) | 23.3 (3.4) | 0.44 |
| Weight (all) | 23 (3.2) | 23.2 (3) | 23.3 (3.4) | 0.51 |
| Female | 22.6 (2.9) | 23.1 (3.1) | 23.1 (3.3) | 0.16 |
| Male | 23.7 (3.6) | 23.6 (2.9) | 23.9 (3.4) | 0.51 |
| Height | 160.3 (8.1) | 158.7 (7.1) | 159.1 (7.9) | 0.009 |
| Female | 156.5 (5.6) | 156.2 (5.4) | 155.9 (5.7) | 0.48 |
| Male | 167.7 (7.2) | 166.8 (5.8) | 168.2 (6) | 0.031 |
| Waist (all) | 75.9 (8.7) | 75.8 (8.2) | 76.2 (9.1) | 0.51 |
| Female | 55.4 (7.6) | 56.4 (8) | 56.1 (8.5) | 0.28 |
| Male | 66.6 (10.2) | 65.8 (9.3) | 67.7 (10.6) | 0.09 |
| Fasting glucose | 91.3 (8.5) | 91 (8.7) | 91.1 (9) | 0.86 |
| Female | 66 | 76 | 74 | 0.007 |
| Impaired fasting glucose | 14 | 14 | 15 | 0.96 |
| LTPA (all), weekly |  |  |  |  |
| <30min | 34 | 36 | 38 |  |
| 30 - 180 min | 31 | 33 | 33 |  |
| >180min | 35 | 30 | 29 |  |
| LTPA (female), weekly |  |  |  |  |
| <30min | 33 | 38 | 41 | 0.26 |
| 30 - 180 min | 32 | 34 | 32 |  |
| >180min | 35 | 28 | 26 |  |
| LTPA (male), weekly |  |  |  |  |
| <30min | 35 | 31 | 29 | 0.75 |
| 30 - 180 min | 30 | 31 | 35 |  |
| >180min | 35 | 38 | 36 |  |
| Family history of diabetes | 26 | 29 | 31 | 0.25 |
| Metabolic syndrome | 12 | 14 | 13 | 0.86 |
| Fatty liver | 47 | 53 | 49 | 0.06 |
| Elevated TG | 21 | 16 | 15 | 0.12 |
| Low HDL-C | 25 | 30 | 27 | 0.13 |
| Vegetarians | 35 | 41 | 37 | 0.08 |
| Female | 42 | 46 | 42 | 0.37 |
| Male | 21 | 27 | 24 | 0.45 |
| Education (all) |  |  |  |  |
| Elementary | 25 | 26 | 20 | <.001 |
| Secondary | 44 | 53 | 50 |  |
| College | 31 | 21 | 30 |  |
| Education (female) |  |  |  |  |
| Elementary | 30 | 28 | 22 | <.001 |
| Secondary | 45 | 55 | 52 |  |
| College | 26 | 17 | 25 |  |
| Education (male) |  |  |  |  |
| Elementary | 17 | 18 | 13 | 0.13 |
| Secondary | 42 | 48 | 42 |  |
| College | 41 | 34 | 44 |  |

**Supplemental Table 2. Sensitivity analysis**

| Analysis | Vegetarians | Converted | Nonvegetarians |
| --- | --- | --- | --- |
| 25 unconfirmed diabetes treated as diabetes | |  |  |
| Model 1 | 0.54 (0.38, 0.76) | 0.45 (0.29, 0.68) | 1 (Reference) |
| Model 2 | 0.65 (0.46, 0.92) | 0.47 (0.30, 0.71) | 1 (Reference) |
| Adjusted for metabolic syndrome in addition to Model 2 | 0.62 (0.44, 0.88) | 0.49 (0.32, 0.75) | 1 (Reference) |
| Only self-reported diabetes were treated as diabetes cases | |  |  |
| Model 1 | 0.56 (0.35, 0.89) | 0.49 (0.28, 0.87) | 1 (Reference) |
| Model 2 | 0.69 (0.43, 1.11) | 0.50 (0.29, 0.89) | 1 (Reference) |
| Age as time scale (truncated by study entry time) | |  |  |
| Model 1 | 0.49 (0.34, 0.69) | 0.45 (0.29, 0.68) | 1 (Reference) |
| Model 2 | 0.60 (0.42, 0.86) | 0.43 (0.28, 0.66) | 1 (Reference) |

Model 1 adjusted for age, gender, education, leisure time physical activities, family history of diabetes, follow-up methods (health examination or questionnaire only), and lipid medication. Model 2 additionally adjusted for BMI.
